# Dermatofibrosarcoma Protuberans of the Forehead: Case Report of a Rare Entity and Review of Literature

**DOI:** 10.1155/crom/9991548

**Published:** 2025-05-08

**Authors:** Ulrich Igor Mbessoh Kengne, Jaafar Ibn Abou Talib Thiam, Amacoumba Fall, Salif Balde, Mamadou Ndiaye, Joel Gabin Konlack Mekontso, Gorgui Sarr, Etienne Tossou Zoure, Mamadou Sow, Sidy Ka

**Affiliations:** ^1^Department of Surgical Oncology, Dalal Jamm National Hospital Center, Dakar, Senegal; ^2^Department of Surgery and Specialties, Faculty of Medicine, Pharmacy and Odontostomatology, Cheikh Anta Diop University, Dakar, Senegal; ^3^Department of Medical Oncology, Dalal Jamm National Hospital Center, Dakar, Senegal; ^4^Department of Internal Medicine, South Brooklyn Hospital, Brooklyn, USA

**Keywords:** dermatofibrosarcoma protuberans, forehead neoplasm, Mohs micrographic surgery, multidisciplinary management, recurrent sarcoma

## Abstract

Dermatofibrosarcoma protuberans (DFSP) is a rare soft tissue sarcoma originating from fibroblasts in the dermal connective tissue, comprising approximately 1% of all soft tissue sarcomas. While most cases involve the trunk and extremities, only 10%–15% occur in the cephalic region, representing less than 1% of all head and neck neoplasms. DFSP is notable for its high propensity for local recurrence following surgical excision and its low metastatic potential. We report a case of recurrent DFSP of the forehead extending to the anterior wall of the left frontal sinus, without brain involvement, in a 33-year-old male with a history of three prior wide local excisions. A multidisciplinary cancer team recommended systemic imatinib therapy. This case highlights the challenges of managing DFSP in an uncommon location, underscoring the importance of a multidisciplinary approach in addressing recurrent and complex presentations.

## 1. Introduction

First described by Darier and Ferrand in 1924 [1, 2], dermatofibrosarcoma protuberans (DFSP) is a rare and indolent cutaneous soft tissue sarcoma originating from fibroblast cells within the dermal connective tissue [3, 4]. Despite its low metastatic potential, DFSP has a significant local recurrence rate of 20%–60% following surgical excision [5, 6]. Accounting for roughly 1% of all soft tissue sarcomas, DFSP predominantly affects the trunk (42%–72%) and extremities (16%–30%) [4, 5, 7], with only 10%–15% of cases localized to the cephalic region and less than 1% involving the head and neck [2, 7]. The forehead is an exceptionally rare site for DFSP, and even fewer cases report involvement of the scalp with skull invasion [8, 9]. Notably, this is the first documented case in West Africa, underscoring both the rarity of the condition and the challenges posed by its location. The forehead's unique anatomy and aesthetic significance introduce complex surgical and psychological considerations, amplifying the therapeutic challenge for oncologists and surgeons. Herein, we report a recurrent DFSP of the forehead with frontal sinus involvement in a 33-year-old male, accompanied by an updated literature review on this topic. This article is presented in accordance with the CARE reporting checklist.

## 2. Case Presentation

A 33-year-old male with a history of poorly controlled epilepsy with frequent breakthrough seizures presented to our institution with a local recurrence of biopsy-proven left frontal scalp DFSP. He had previously undergone wide local excision (WLE) of the tumor on three occasions (2016, 2019, and 2020) at an outside institution, with histological and immunohistochemical studies consistently confirming the diagnosis of DFSP. No adjuvant therapy had been administered following these surgeries.

**Figure 1:**
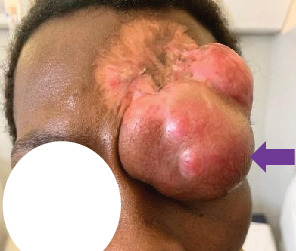
Brownish-red, multilobulated mass growing on the scar of the previous WLE (frontal view).

**Figure 2:**
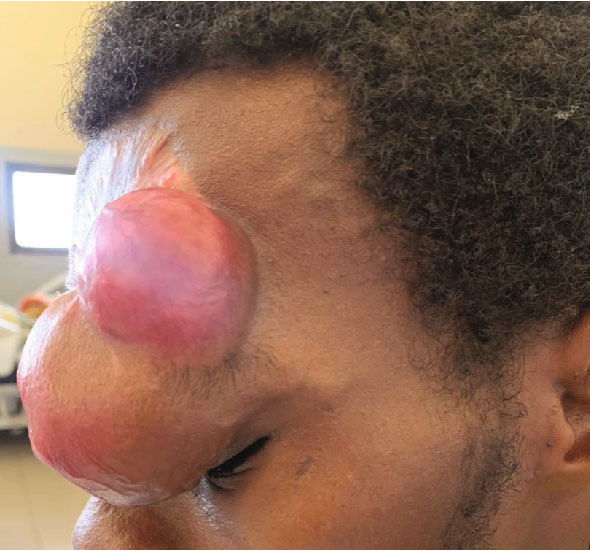
Brownish-red, multilobulated mass growing on the scar of the previous WLE (lateral view).

On presentation, the patient was vitally stable and exhibited a solitary, brownish-red, firm, multilobulated nodule on the left forehead, measuring approximately 7 × 5 cm (Figures 1 and 2). The mass extended to the ipsilateral eyelid was fixed to the underlying tissues and had arisen over the scar from previous WLE. There was an ipsilateral palpebral ptosis and complete eye occlusion. The lesion was nontender and nonpulsatile and did not exhibit transillumination. The rest of the physical exam was unremarkable.

**Figure 3:**
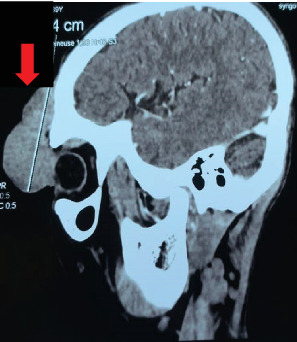
Contrast-enhanced head CT (sagittal view) showing a well-defined, enhancing soft tissue mass in the left frontal region, measuring 64 × 56 × 32 mm (red arrow). The lesion is associated with underlying bone lysis involving the anterior wall of the frontal sinus.

**Figure 4:**
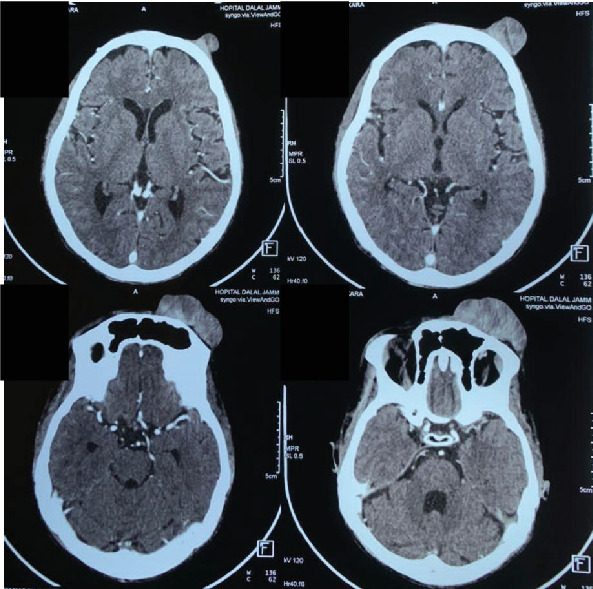
Contrast-enhanced head CT (axial view) showing no evidence of brain involvement by the tumor.

Head computed tomography (CT) with intravenous contrast showed significant enhancement of the mass, along with bone lysis involving the anterior wall of the frontal sinus. Importantly, no brain invasion was observed (Figures 3 and 4). A metastatic workup revealed no evidence of distant spread.

Given the tumor's unresectable nature, the multidisciplinary cancer team (MCT) recommended initiating imatinib-based systemic therapy for 18 months, followed by a reassessment for potential surgical intervention. Currently, the patient is undergoing imatinib therapy and is being closely monitored for response in anticipation of future surgical resection.

## 3. Discussion

DFSP is an uncommon, locally aggressive cutaneous sarcoma typically characterized by a t(17;22)(q22;q13) translocation [10, 11]. This translocation results in the formation of a fusion gene between the collagen type I alpha 1 (COL1A1) and platelet-derived growth factor beta (PDGFB) genes. The resulting overexpression of PDGFB promotes tumorigenesis through enhanced cell proliferation and survival [4, 10]. Interestingly, this patient has a history of epilepsy with frequent breakthrough seizures, a potential source of repetitive trauma. Trauma has been identified as a possible contributing factor in 10%–30% of DFSP cases, as highlighted by various case series [5, 12]. Reported traumatic events include injections, tattoos, central line catheterization, drainage tube insertions, surgical scars, burns, and road traffic accidents [5, 12]. Despite these observations, the causal relationship and underlying mechanisms connecting trauma and DFSP remain unclear [12].

DFSP is most commonly encountered in adults between the ages of 30 and 50, affecting both genders [4, 6]. However, some series suggest a slightly higher prevalence in men [5, 10]. These epidemiological observations align with our case. It predominantly occurs on the trunk, followed by the extremities, and is less commonly found on the head and neck region, including the forehead [5, 8]. Macroscopically, DFSP presents as either an irregular protuberant swelling or a hard, indurated plaque. The lesion typically grows slowly and may exhibit violaceous, blue–red, or brown discoloration [4, 5]. Over time, it progressively invades surrounding tissues, including fat, fascia, muscles, periosteum, and neurovascular structures [13]. In this patient, the tumor demonstrated aggressive infiltration, extending into the sinus of the frontal bone. Although metastasis in DFSP is rare, when it does occur, the metastatic cells predominantly target the lungs, brain, bones, and abdominal viscera [4].

The differential diagnosis of DFSP is complex and evolves with tumor growth; in its early stages, this often presents a diagnostic challenge as DFSP can closely mimic a range of benign conditions, including lipomas, epidermal cysts, keloids, hypertrophic scars, dermatofibromas, or nodular fasciitis. DFSP in unusual locations like the forehead can also be misdiagnosed as congenital or early-onset lesions such as dermoid cysts or vascular malformations due to its slow growth and variable presentation (e.g., plaques or nodules with reddish or purplish hues). A high index of suspicion and biopsy remain crucial for accurate differentiation. At later stages, DFSP may resemble more serious conditions such as pyogenic granuloma, Kaposi sarcoma, schwannoma, neurofibroma, basal cell carcinoma, or other soft tissue sarcomas [5, 6].

The diagnostic workup for suspected cutaneous malignancy begins with obtaining a punch or excisional biopsy specimen for histological analysis [4]. Microscopically, DFSP is characterized by a cartwheel or storiform pattern of spindle-shaped tumor cells. A hallmark feature is its infiltration into the subcutaneous tissue in a typical finger-like or honeycomb pattern [2, 4–7]. Immunohistochemical studies further aid in diagnosis, revealing strong positivity for CD34 and vimentin, with negativity for factor XIIIa, S-100, and CD44 [2, 4–7]. In challenging cases, fluorescence in situ hybridization (FISH) can detect the hallmark t(17;22)(q22;q13) translocation, confirming the COL1A1-PDGFB fusion gene. This molecular alteration is frequently identified in over 90% of DFSP cases [2, 5, 10].

When diagnosing or confirming DFSP, MRI is the preferred modality for evaluating the extent of local tumor invasion due to its superior resolution for soft tissue differentiation [4]. However, in cases where DFSP arises on the head, the limited amount of soft tissue overlying the bone often leads to MRI underestimating the true depth of tumor infiltration [12]. In such scenarios, CT becomes a valuable alternative, offering higher sensitivity for detecting cortical bone invasion [12]. In our case, CT imaging was critical in demonstrating the extension of the tumor to the anterior wall of the frontal sinus.

Despite the utility of these imaging modalities, the radiological features of DFSP are relatively nonspecific. On both CT and MRI, DFSP typically presents as a subcutaneous, well-demarcated soft tissue mass with moderate to significant contrast enhancement [2].

Hao et al. recently proposed a modified staging system for DFSP, which is derived from European consensus guidelines and incorporates the tumor's pathological features and metastatic extent [14]. This system could potentially enhance clinical decision-making and prognostic assessments.

Management of DFSP of the forehead often necessitates a multidisciplinary approach involving head and neck surgeons, surgical oncologists, plastic surgeons, neurosurgeons, radiation oncologists, and, in advanced cases, medical oncologists [13, 15]. Literature suggests that surgical intervention remains the cornerstone of treatment for DFSP of the forehead, with the primary objective being the achievement of clear resection margins [4, 16]. Two commonly performed surgical procedures for early-stage DFSP are WLE and Mohs micrographic surgery (MMS) (see Table 1). Given the cosmetic and functional importance of the forehead, surgical planning requires careful consideration of reconstructive options, ranging from primary closure for smaller defects to local or free flaps for larger excisions, aiming for both oncological control and optimal aesthetic outcomes.

In WLE, the tumor, along with the underlying muscle fascia, is entirely excised, typically aiming for tumor-free margins of 2–4 cm [4, 16]. This approach frequently necessitates subsequent immediate or delayed reconstruction. MMS, in contrast, involves the sequential removal and microscopic examination of thin tissue layers until no cancer cells remain [4], offering the advantage of minimizing safety margins to approximately 1 cm with immediate margin assessment and revision, often making it the preferred surgical approach [17]. Beyond standard histological assessment of surgical margins, techniques like FISH, capable of detecting the *COL1A1-PDGFB* fusion gene, may offer a more sensitive approach to identifying residual neoplastic cells that might be missed by conventional methods. Hallier et al. [17] highlighted the potential of FISH in more accurately defining tumor-free margins, potentially reducing local recurrence rates and guiding more targeted postoperative therapies.

Achieving a 3-cm margin of cancer-free tissue in the forehead region can be particularly challenging due to the critical functional and cosmetic considerations inherent to this anatomical site [7]. To address these limitations, radiotherapy has emerged as a valuable adjuvant to WLE, particularly when surgical margins are positive or inadequate for disease control [5].

DFSP with skull invasion is a rare entity with limited data guiding optimal management [9]. To inform our approach, we reviewed the literature and summarized previously published cases of DFSP, including locally advanced cases with bone involvement, in Table 1. For skull involvement, the National Comprehensive Cancer Network (NCCN) guidelines recommend imatinib, particularly for unresectable tumors, metastatic disease, recurrence, or when surgery carries unacceptable functional or cosmetic risks [13]. Consistent with these recommendations, we initiated imatinib therapy in our patient with unresectable recurrent DFSP. Postoperative adjuvant therapy, such as radiation or systemic agents like imatinib, may be considered based on the extent of disease and resection margins. Given the patient's history of poorly controlled epilepsy, careful monitoring for potential drug–drug interactions, especially considering the shared metabolic pathways of imatinib and many antiepileptic medications, is crucial, along with strategies to support adherence to both treatment plans to mitigate the risk of subtherapeutic drug levels or increased toxicity.

DFSP is known for its high recurrence rates [3, 4]. Studies report local recurrence rates ranging from 20% to 75% following surgery, with the head and neck region exhibiting the highest rates of recurrence, ranging from 50% to 75% [2, 4, 9]. This aligns with our patient's medical history, which revealed three local recurrences of the tumor over a 5-year period. Given the high risk of local recurrence, long-term follow-up with regular clinical examinations and potential imaging is crucial for early detection of any recurrence.

**Figure 5:**
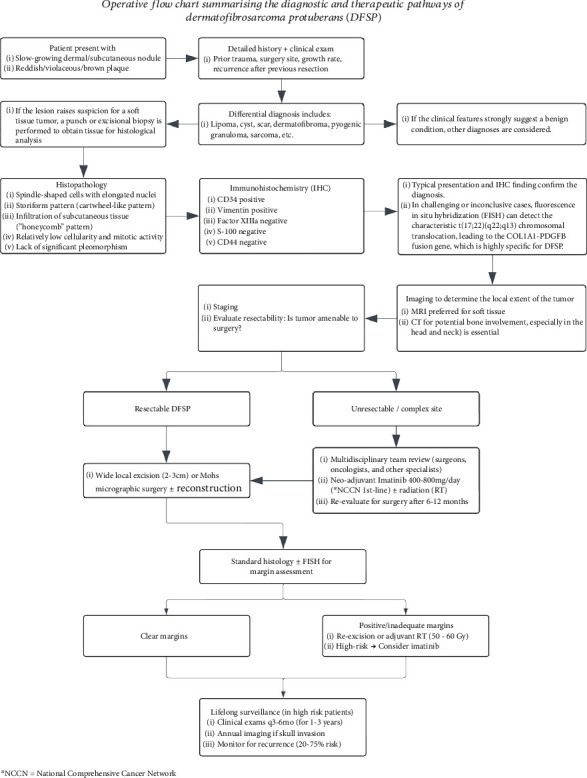
Operative flow chart summarizing the diagnostic and therapeutic pathways of dermatofibrosarcoma protuberans.

Evidence suggests that, irrespective of tumor location, DFSP has a nearly 50% risk of local recurrence within the first year following initial treatment, rising to 80% within 3 years [5]. The primary cause of recurrence is often attributed to the incomplete excision of clinically unapparent projections of the tumor [5]. This underscores the importance of meticulous surgical planning and the potential utility of adjunctive therapies to minimize recurrence risk.  Figure 5 illustrates the operative flowchart, summarizing the diagnostic workup and therapeutic decision-making process for DFSP.

## 4. Conclusion

DFSP is a rare cutaneous sarcoma, infrequently observed in the head and neck region. A hallmark of this malignancy is its pronounced propensity for local recurrence. WLE is considered appropriate for treating resectable tumors, aiming to achieve clear margins while minimizing the risk of recurrence. In cases of recurrence, unresectable tumors, or when surgical excision would result in significant functional or cosmetic impairment, targeted therapy with imatinib emerges as the preferred treatment option. Effective management of DFSP necessitates a multidisciplinary approach, involving collaboration among oncologists, surgeons, and other specialists, underscoring the complexity and challenges associated with treating this rare entity. Long-term follow-up is essential due to the significant risk of local recurrence.

## Figures and Tables

**Table 1:** A review of previously published DFSP of the forehead and scalp.

**Authors**	**Year**	**Age**	**Gender**	**Past history**	**Report of recurrence**	**Tumor extension**	**Treatment**
Aleem et al. [6]	2023	48	Male	No	No	Localized	WLE
Benoît et al. [8]	2016	53	Male	No	No	Localized	MMS
Benoît et al. [8]	2016	36	Female	Trauma	Yes	Localized	MMS
Khachemoune et al. [1]	2006	32	Female	No	No	Locally advanced	MMS
Mani et al. [13]	2022	47	Male	No	Yes	Localized	WLE
Mani et al. [13]	2022	47	Female	No	Yes	Localized	WLE
Mori et al. [7]	2012	52	Male	No	No	Localized	WLE
Mori et al. [7]	2012	45	Male	No	Yes	Localized	WLE
Rahman et al. [2]	2023	40	Female	No	Yes	Localized	WLE
Rahman et al. [2]	2023	48	Male	No	No	Locally advanced	WLE
Redondo et al. [16]	2022	27	Male	No	No	Locally advanced	MMS
Steele et al. [12]	2020	32	Male	Trauma	No	Locally advanced	WLE
Wuennenberg et al. [9]	2023	49	Male	No	Yes	Locally advanced	MMS
